# Numerical Simulation of Mixed Convection Squeezing Flow of a Hybrid Nanofluid Containing Magnetized Ferroparticles in 50%:50% of Ethylene Glycol–Water Mixture Base Fluids Between Two Disks With the Presence of a Non-linear Thermal Radiation Heat Flux

**DOI:** 10.3389/fchem.2020.00792

**Published:** 2020-09-23

**Authors:** Kottakkaran Sooppy Nisar, Umair Khan, A. Zaib, Ilyas Khan, Dumitru Baleanu

**Affiliations:** ^1^Department of Mathematics, College of Arts and Science, Prince Sattam Bin Abdulaziz University, Wadi Al Dawasir, Saudi Arabia; ^2^Department of Mathematics and Social Sciences, Sukkur IBA University, Sukkur, Pakistan; ^3^Department of Natural Sciences, The Begum Nusrat Bhutto Women University, Sukkur, Pakistan; ^4^Department of Mathematical Sciences, Federal Urdu University of Arts, Science & Technology, Karachi, Pakistan; ^5^Faculty of Mathematics and Statistics, Ton Duc Thang University, Ho Chi Minh City, Vietnam; ^6^Department of Mathematics, Cankaya University, Ankara, Turkey; ^7^Institute of Space Sciences, Măgurele, Romania; ^8^Department of Medical Research, China Medical University Hospital, China Medical University, Taichung City, Taiwan

**Keywords:** squeeze flow, mixed convection, magnetize hybrid ferrofluids, non-linear radiation, disks

## Abstract

Ferroliquids are an example of a colloidal suspension of magnetic nanomaterials and regular liquids. These fluids have numerous applications in medical science such as cell separation, targeting of drugs, magnetic resonance imaging, etc. The hybrid nanofluid is composed by scattering the magnetic nanomaterial of more than one type nanoparticles suspended into the base fluid. It has different scientific applications such as heat dissipation, dynamic sealing, damping, etc. Owing to the vast ferrofluid applications, the time-dependent squeezed flow of hybrid ferroliquids under the impact of non-linear radiation and mixed convection within two disks was explored for the first time in this analysis. Here, the cobalt and magnetite ferrofluids are considered and scattered in a 50%:50% mixture of water–EG (ethylene glycol). The similarity technique is used to reduce the leading PDEs into coupled non-linear ODEs. The transmuted equations together with recommended boundary restrictions are numerically solved via Matlab solver bvp4c. The opposing and assisting flows are considered. The impacts of an emerging parameter on fluid velocity and temperature field of hybrid ferroliquids are examined through the different graphical aids. The results showed that the opposite trend is scrutinized due to the magnetic influence on the temperature and velocity in the case of assisting and opposing flows. The velocity augments due to the volume fraction of nanoparticles in the assisting flow and declines in the opposing flow, while the opposite direction is noticed in the temperature field.

## Introduction

The time-dependent squeezed flow is regularly addressed in realistic surroundings to describe the movement of fluid along an area of contracting of a recommended length. This flow can be set up via placing a preliminary sluggish liquid between a couple of rectangular parallel disks and plates to indicate the related mathematical models on specific planes of coordinates. The research interest in studying the squeezed flows is quickly developed owing to existing and rising applications in polymer processes of manufacturing and biological liquid transport, purification, lubrication, molding injection, filtration, etc. Ishizawa (1966) inspected the motion of squeezed flow by unstable distance in moving two disks and employed the perturbation technique to find the solution. An exact solution of a similar problem in a couple of elliptic plates with the multifold time-dependent series was obtained by Usha and Sridharan (1996). Duwairi et al. (2004) explored the time-dependent viscous squeezing flow between parallel two plates with a constant temperature. Domairry and Aziz (2009) applied the magnetic function on squeezing flow between two disks. The magnetic function on squeezing flow connecting two analogous permeable disks was inspected by Hayat et al. (2012a). Since then, many researchers have contributed to the progress of squeezed flow for distinct models of fluid (Hayat et al., 2012b; Qayyum et al., 2012; Sheikholeslami and Ganji, 2013; Sheikholeslami et al., 2013; Domairry and Hatami, 2014). In recent times, Hayat et al. (2018a,b) discussed the squeezed flow comprising carbon nanotubes through two parallel disks in a Darcy–Forchheimer medium with thermal radiation. Alzahrani et al. (2020) analyzed the squeezed 3D flow through a rotated in a channel with Dufour and Soret impacts. The impact of imposed magnetic forces on squeezed flow containing nanomaterials between two plates was examined by Babazadeh et al. (2020).

The exploration of nanofluids has acknowledged a prominent interest owing to their incredible range of applications involving the processing of nanomaterials, the coolant of automobiles, polymer coating, tribology of aerospace, and suspensions of medical sterilization. Choi (1995) coined the term “nanofluid” for the first time, which refers to the submicron scattering of solid material into a liquid, having greater thermal conductivity in a convectional liquid. It is perceptible that these nanomaterials are considered ultrafine (1–50 nm order of length); hence, the nanoliquids appeared to conduct further as a single-phase liquid than a suspension of solid–liquid. Comprehensive monographs regarding the nanofluids in conjunction with several applications can be seen in Tiwari and Das (2007), Chamkha et al. (2012), El-Kabeir et al. (2015), Wakif et al. (2018), Sheikholeslami (2019), Soomro et al. (2019), Mebarek-Oudina et al. (2020), and Raza et al. (2020). More precisely, the magnetic nanoliquid (ferroliquids), which is colloidally deferred of magnetic nanomaterials like ferrite, cobalt, and magnetite, is suspended in non-conducting fluids like heptanes, water, hydrocarbons, and kerosene. These ferrofluids have numerous applications in medical science like cell separation, targeting of drug medicine, imaging of magnetic resonance, etc. Neuringer (1996) inspected the thermal gradient and magnetic function on the flow of ferrofluids. The impact of uniform heat flux on slip flow with heat transport from a flat surface was analyzed by Khan et al. (2015). They considered three different ferrofluids (CoFe_2_O_4_, Fe_3_O_4_, and Mn-ZnFe_2_O_4_) with two different base fluids (water and kerosene). Rashad (2017) scrutinized the magnetic function on slip flow containing kerosene-based cobalt ferrofluid from a non-isothermal wedge with mixed convection and radiation effect. Zaib et al. (2019) inspected the entropy on mixed convective flow comprising the magnetite ferroliquid of micropolar fluid from a vertical plate. Recently, Ali et al. (2020) discussed the micropolar fluid with magnetic dipole impact comprising EG and water-based ferrofluids, namely, Fe and Fe_3_O_4_ from a stretched sheet. On the other hand, hybrid nanoliquids are deliberate to catch superior properties of thermal as well as rheological via the mixing couple of distinct nanoparticles. A reason following the initiation of hybrid ferroliquids is to knob the heat transport efficiency in the fluid flow. It has different scientific applications as dynamic sealing, naval, damping, acoustics, microfluidics, etc. The impact of dissipation on time-dependent flow comprising hybrid nanoliquid through a rounded pipe was examined by Suresh et al. (2012). They accomplished that nanoliquid has a lower friction factor than hybrid nanoliquid. Madhesh and Kalaiselvam (2015) discussed the pressure drop, in which the Reynolds and Peclet numbers are investigated for the volume fraction of water-based Cu-TiO_2_ hybrid nanoliquid. Adriana (2017) presented the correlation of alumina hybrids and nanofluids through the date of the temperature gradient. The features of fluid flow of water-based CuO-Ag hybrid nanoliquids were inspected by Hayat and Nadeem (2017) under the impacts of chemical reaction and radiation. Mebarek-Oudina (2019) discussed the characteristics of thermal and hydrodynamic of Titania nanoliquids satisfying a cylindrical annulus. Mahanthesh et al. (2019) analyzed the influences of an exponential space-dependent heat source on magneto slip flow comprising carbon nanofluids from a stretchable rotating disk. Wakif et al. (2020) explored the influences of surface roughness and radiation containing the stability of Al_2_O_3_-CuO hybrid nanoliquid by employing the generalized Buongiorno model. Recently, Marzougui et al. (2020) investigated the entropy analysis through convective flow involving nanoliquid via MHD with chamfers in a cavity.

The scrutiny of the magnetic function has considerable solicitations in MHD bearings; geology, astrophysics, pumps, generators, medicine, control of boundary layer, etc. are few prominent magneto-hydrodynamics applications. Alshomrani and Gul (2017) examined the thin-film flow of water-based Al_2_O_3_ and Cu nanofluid through a stretched cylinder under the impact of magnetic function. Sandeep and Malvandi (2016) investigated the features of magneto thermal transport containing the time-dependent flow of liquid movement of the nanofluid thin film of a starched surface. The features of hybrid nanoliquid flow with variable heat and drag forces were explored by Sandeep (2017). Ahmad and Nadeem (2017) scrutinized the magnetic impact on micropolar fluid comprising hybrid nanofluid with a heat sink/source and obtained the multiple results for hybrid nanofluid and micropolar fluid. Hamrelaine et al. (2019) investigated the magnetic impact of Jeffery-Hamel flow between non-parallel permeable walls or permeable plates. The magnetic effect on the radiative flow of the hybrid nanoliquid thin film with irregular heat sink/source was inspected by Kumar et al. (2020). Zaib et al. (2020) obtained the similarity of multiple results from magnetite ferroliquid conveying non-Newtonian blood flow with entropy generation. Wakif et al. (2019) estimated the impact of the magnetic field on flows of Stokes' second problem with entropy generation. Recently, Wakif (2020) examined the magneto flow of Casson blood flow from a horizontal stretched surface with rough geometry, thermal conductivity, and temperature viscosity.

As the literature discussed above, the non-linear radiative flow of hybrid ferroliquids from a squeezed mixed convective flow within two disks has not yet been explored. Thus, this investigation's core objective is to examine the non-linear radiative magneto flow of hybrid ferrofluids in a squeezed flow within two disks. Such sort of problems is created by biomedical enforcement and electric generators. The hybrid nanoparticles have constructive biomedical applications like cancer therapy, drug delivery, biosensing, etc. The liquid model is altered into ODEs through acceptable technique and investigated numerically through the Lobatto 3A formula.

## Problem Formulation

**Figure 1:**
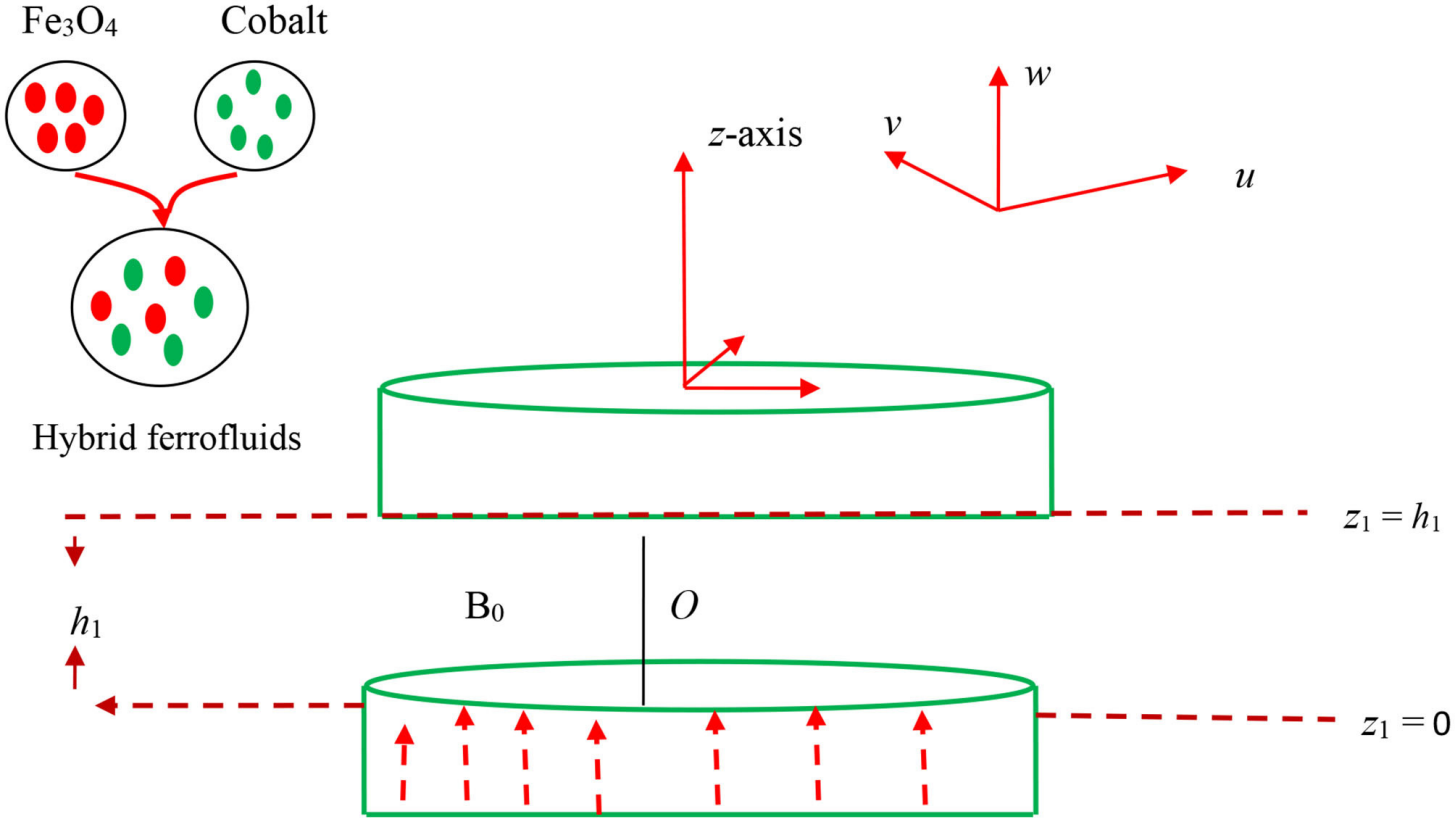
Physical diagram of the problem.

Figure 1 demonstrates the problem's physical background of the flow configuration schematically. Consider a 2D magneto-hydrodynamic (MHD), incompressible, and squeezing flow of a hybrid ferrofluid along with axisymmetric and time-dependent flow between the two infinite parallel disks alienated by a distance $h_{1}\left(t\right)=H_{1}{\left(1-bt\right)}^{0.5},$ where *b* is called the characteristic parameter whose dimension is *t*^−1^. Further, interpretation of this point shows that the squeezing movements of both the disk are occurring in the case *b* > 0 with velocity *w*_1_ = *dh*_1_/*dt* until they touch each other at *bt* = 1 while the case *b* < 0 refers to the movement of the disk away to each other. The initial or starting point of the disk is represented by *H*_1_ at time *t* = 0. The regular liquid is caught to be 50%:50% (ethylene glycol + water). The time-dependent magnetic field is proportional to $B_{1}\left(t\right)=B_{0}/{\left(1-bt\right)}^{0.5}$ and is enforced normally to the disks. Also, the field of induced magnetic field is ignored owing to the assumption of small Reynold's number. The influences of mixed convective and thermal radiation are invoked in the governing equations. *T*_*w*_ refers to the constant temperature of the lower disk *z*_1_ = 0, while *T*_*h*_1__ called the constant temperature of the upper disk located at *z*_1_ = *h*_1_ (*t*). The upper disk located at *z*_1_ = *h*_1_ (*t*) is moving away or toward with the velocity $0.5bH_{1}{\left(1-bt\right)}^{0.5}$ from the stationary lower disk at *z*_1_ = 0. Based on these suppositions, the prevailing constitutive equations of the momentum and energy for the time-dependent viscous hybrid ferrofluid are (Hayat et al., 2012a, 2018a; Babazadeh et al., 2020).

(1) $$\begin{array}{l} \frac{\partial w_{1}}{\partial z_{1}}+\frac{u_{1}}{r_{1}}+\frac{\partial u_{1}}{\partial r_{1}}=0, \end{array}$$

(2) $$\begin{array}{l} \frac{\partial u_{1}}{\partial t}+w_{1}\frac{\partial u_{1}}{\partial z_{1}}+u_{1}\frac{\partial u_{1}}{\partial r_{1}}\\ \;=-\frac{\sigma_{hnf}B_{1}^{2}\left(t\right)}{\rho_{hnf}}u_{1}+\frac{\mu_{hnf}}{\rho_{hnf}}\left(\frac{\partial^{2}u_{1}}{\partial {r_{1}}^{2}}+\frac{1}{r_{1}}\frac{\partial u_{1}}{\partial r_{1}}+\frac{\partial^{2}u_{1}}{\partial {z_{1}}^{2}}-\frac{u_{1}}{{r_{1}}^{2}}\right)\\ \;+\frac{g{\left(\rho \beta \right)}_{hnf}}{\rho_{hnf}}\left(T_{1}-T_{h_{1}}\right)-\frac{1}{\rho_{hnf}}\frac{\partial p}{\partial r_{1}}, \end{array}$$

(3) $$\begin{array}{l} \frac{\partial w_{1}}{\partial t}+w_{1}\frac{\partial w_{1}}{\partial z_{1}}+u_{1}\frac{\partial w_{1}}{\partial r_{1}}\\ \;=-\frac{1}{\rho_{hnf}}\frac{\partial p}{\partial z_{1}}+\frac{\mu_{hnf}}{\rho_{hnf}}\left(\frac{\partial^{2}w_{1}}{\partial {r_{1}}^{2}}+\frac{1}{r_{1}}\frac{\partial w_{1}}{\partial r_{1}}+\frac{\partial^{2}w_{1}}{\partial {z_{1}}^{2}}\right), \end{array}$$

(4) $$\begin{array}{l} {\left(\rho c_{p}\right)}_{hnf}\left(\frac{\partial T_{1}}{\partial t}+u_{1}\frac{\partial T_{1}}{\partial r_{1}}+w_{1}\frac{\partial T_{1}}{\partial z_{1}}\right)\\ \;=k_{hnf}\left(\frac{\partial^{2}T_{1}}{\partial {r_{1}}^{2}}+\frac{1}{r_{1}}\frac{\partial T_{1}}{\partial r_{1}}+\frac{\partial^{2}T_{1}}{\partial {z_{1}}^{2}}\right)+\frac{16\sigma^{*}}{3k^{*}}\frac{\partial }{\partial z_{1}}\left({T_{1}}^{3}\frac{\partial T_{1}}{\partial z_{1}}\right) \end{array}$$

with the boundary conditions (Babazadeh et al., 2020)

(5) $$\begin{array}{l} \begin{array}{c} z_{1}=0:u_{1}=0,\;w_{1}=-\frac{w_{0}}{\sqrt{1-bt}},\;T_{1}=T_{w},\\ z_{1}=h_{1}\left(t\right):u_{1}=0,\;w_{1}=\frac{dh_{1}}{dt},\;T_{1}=T_{h_{1}}. \end{array} \end{array}$$

where the pressure and temperature of the flow of fluid are represented by the symbols *p* and *T*_1_, respectively. The rest of the mathematical notions and letters of symbols in the Equations (1)–(6) are defined in the nomenclature, while the thermophysical properties of the normal fluid and the hybrid ferrofluid are shown in Table 1.

**Table 1:** Properties of thermophysical of normal liquid and ferroparticles (Rashad, 2017; Kumar et al., 2020).

**Physical properties**	***H*_2_*O* + *EG* 50%**	***Fe*_3_*O*_4_**	**Cobalt**
*c*_*p*_(J/kg K)	3,288	670	420
ρ(kg/m^3^)	1,056	5,180	8,900
*k*(W/mK)	0.425	9.7	100
β × 10^−5^(1/K)	0.00341	1.3	1.3 × 10^−5^
σ(Sm^−1^)	0.00509	0.74 × 10^6^	1.602 × 10^7^
Pr	29.86		

In addition, the mathematical expressions of the studied hybrid ferrofluid are given by:

(6) $$\begin{array}{l} \left\{\begin{array}{c} \rho_{hnf}=\left(1-\phi \right)\rho_{f}+\phi_{1s}\rho_{1s}+\phi_{2s}\rho_{2s},\;\mu_{hnf}=\frac{\mu_{f}}{{\left(1-\phi \right)}^{2.5}},\\ \;\phi =\phi_{1s}+\phi_{2s}\text{,}\\ \sigma_{hnf}/\sigma_{f}\\ \;=\left\{1+\frac{3\left(\frac{\sigma_{1s}\phi_{1s}+\sigma_{2s}\phi_{2s}}{\sigma_{f}}-\phi \right)}{\left(\frac{\sigma_{1s}+\sigma_{2s}}{\sigma_{f}}+2\right)-\left(\frac{\sigma_{1s}\phi_{1s}+\sigma_{2s}\phi_{2s}}{\sigma_{f}}\right)+\phi }\right\}\text{,}\\ {\left(\rho \beta \right)}_{hnf}=\left(1-\phi \right){\left(\rho \beta \right)}_{f}+\phi_{1s}{\left(\rho \beta \right)}_{1s}+\phi_{2s}{\left(\rho \beta \right)}_{2s}\text{,}\\ \frac{k_{hnf}}{k_{f}}=\frac{\left(k_{1s}+k_{2s}\right)+2\left(1-\phi \right)k_{f}+2\phi_{1s}k_{1s}+2\phi_{2s}k_{2s}}{\left(k_{1s}+k_{2s}\right)+\left(2+\phi \right)k_{f}-\left(\phi_{1s}k_{1s}+\phi_{2s}k_{2s}\right)},\\ \alpha_{hnf}=k_{hnf}/{\left(\rho c_{p}\right)}_{hnf},{\left(\rho c_{p}\right)}_{hnf}=\left(1-\phi \right){\left(\rho c_{p}\right)}_{f}\\ \;+\;\phi_{1s}{\left(\rho c_{p}\right)}_{1s}+\phi_{2s}{\left(\rho c_{p}\right)}_{2s}, \end{array}\right\} \end{array}$$

where ϕ is the total volume fraction of ferroparticles. The volume fractions of the individual ferroparticles are indicated by the symbol ϕ_1_ and ϕ_2_, while the subscripts *hnf, f*, and *s* represent the hybrid ferrofluid, base fluid, and solid particles. Further, to ease this analysis, the similarity variables are introduced as the following

(7) $$\begin{array}{l} \begin{array}{c} u_{1}=\frac{0.5}{1-bt}br_{1}F^{\prime }\left(\eta \right),w_{1}=-\frac{H_{1}}{\sqrt{1-bt}}bF\left(\eta \right),B_{1}\left(t\right)=\frac{B_{0}}{\sqrt{1-bt}}\\ T=T_{h_{1}}\left[1+\left(\theta_{h_{1}}-1\right)\theta \right]\text{,}\eta =\frac{z_{1}}{H_{1}\sqrt{1-bt}}. \end{array} \end{array}$$

Now, using the aforementioned transformation along with Equation (6), the governing PDEs, Equations (2) to (4), along with the boundary condition (5) becomes

(8) $$\begin{array}{l} \frac{1}{\mu_{hnf}}\left(\frac{2{H_{1}}^{2}{\left(1-bt\right)}^{2}}{br_{1}}\right)\frac{\partial p}{\partial r_{1}}\\ =F^{\prime \prime \prime }-\frac{S\frac{\rho_{hnf}}{\rho_{f}}}{\frac{\mu_{hnf}}{\mu_{f}}}\left(2F^{\prime }+\eta F^{\prime \prime }+{F{}^{\prime }}^{2}-2FF^{\prime \prime }\right)\\ -\frac{\frac{\sigma_{hnf}}{\sigma_{f}}M^{2}}{\frac{\mu_{hnf}}{\mu_{f}}}f^{\prime }+\frac{\lambda \frac{{\left(\rho \beta \right)}_{hnf}}{{\left(\rho \beta \right)}_{f}}}{\frac{\mu_{hnf}}{\mu_{f}}}\theta \end{array}$$

(9) $$\begin{array}{l} \frac{1}{\mu_{hnf}}\left(\frac{H_{1}{\left(1-bt\right)}^{\frac{3}{2}}}{b}\right)\frac{\partial p}{\partial z_{1}}=-F^{\prime \prime }-\frac{S\frac{\rho_{hnf}}{\rho_{f}}}{\frac{\mu_{hnf}}{\mu_{f}}}\left(F+\eta F^{\prime }-2FF^{\prime }\right) \end{array}$$

(10) $$\begin{array}{l} \theta^{\prime \prime }\left(\frac{k_{hnf}}{k_{f}}+\frac{4}{3}R_{d}{\left(1+\left(\theta_{h_{1}}-1\right)\theta \right)}^{3}\right)\\ +\;4R_{d}{\left(1+\left(\theta_{h_{1}}-1\right)\theta \right)}^{2}\left(\theta_{h_{1}}-1\right){\theta {}^{\prime }}^{2}\\ -\;\mathrm{Pr}S\frac{{\left(\rho c_{p}\right)}_{hnf}}{{\left(\rho c_{p}\right)}_{f}}\left(\eta \theta^{\prime }-2F\theta^{\prime }\right)=0, \end{array}$$

Eliminating the pressure term from Equations (8) and (9), the final form of the momentum and energy equation becomes

(11) $$\begin{array}{l} F^{\prime \prime \prime \prime }-\frac{SA_{1}}{A_{2}}\left(3F^{\prime \prime }+\eta F^{\prime \prime \prime }-2FF^{\prime \prime \prime }\right)-\frac{A_{3}M^{2}}{A_{2}}F^{\prime \prime }+\frac{\lambda A_{4}}{A_{2}}\theta^{\prime }=0, \end{array}$$

(12) $$\begin{array}{l} \theta^{\prime \prime }\left(A_{5}+\frac{4}{3}R_{d}{\left(1+\left(\theta_{h_{1}}-1\right)\theta \right)}^{3}\right)\\ \;+\;4R_{d}{\left(1+\left(\theta_{h_{1}}-1\right)\theta \right)}^{2}\left(\theta_{h_{1}}-1\right){\theta {}^{\prime }}^{2}\\ \;-\;\mathrm{Pr}SA_{6}\left(\eta \theta^{\prime }-2F\theta^{\prime }\right)=0, \end{array}$$

with the boundary restrictions

(13) $$\begin{array}{l} \begin{array}{c} F\left(0\right)=A,F^{\prime }\left(0\right)=0,\theta \left(0\right)=1,\\ F\left(1\right)=\frac{1}{2},F^{\prime }\left(1\right)=0,\theta \left(1\right)=0. \end{array} \end{array}$$

In which:

$$\begin{array}{l} A_{1}=\frac{\rho_{hnf}}{\rho_{f}}=\left(1-\phi \right)+\phi_{1s}\left(\frac{\rho_{1s}}{\rho_{f}}\right)+\phi_{2s}\left(\frac{\rho_{2s}}{\rho_{f}}\right),\\ A_{2}=\frac{\mu_{hnf}}{\mu_{f}}=\frac{1}{{\left(1-\phi \right)}^{2.5}},\phi =\phi_{1s}+\phi_{2s},\\ A_{3}=\frac{\sigma_{hnf}}{\sigma_{f}}=\left\{1+\frac{3\left(\frac{\sigma_{1s}\phi_{1s}+\sigma_{2s}\phi_{2s}}{\sigma_{f}}-\phi \right)}{\left(\frac{\sigma_{1s}+\sigma_{2s}}{\sigma_{f}}+2\right)-\left(\frac{\sigma_{1s}\phi_{1s}+\sigma_{2s}\phi_{2s}}{\sigma_{f}}\right)+\phi }\right\},\\ A_{4}=\frac{{\left(\rho \beta \right)}_{hnf}}{{\left(\rho \beta \right)}_{f}}=\left(1-\phi \right)+\phi_{1s}\left(\frac{{\left(\rho \beta \right)}_{1s}}{{\left(\rho \beta \right)}_{f}}\right)+\phi_{2s}\left(\frac{{\left(\rho \beta \right)}_{2s}}{{\left(\rho \beta \right)}_{f}}\right),\\ A_{5}=\frac{k_{hnf}}{k_{f}}=\frac{\left(k_{1s}+k_{2s}\right)+2\left(1-\phi \right)k_{f}+2\phi_{1s}k_{1s}+2\phi_{2s}k_{2s}}{\left(k_{1s}+k_{2s}\right)+\left(2+\phi \right)k_{f}-\left(\phi_{1s}k_{1s}+\phi_{2s}k_{2s}\right)},\\ A_{6}=\frac{{\left(\rho c_{p}\right)}_{hnf}}{{\left(\rho c_{p}\right)}_{f}}=\left(1-\phi \right)+\phi_{1s}\left(\frac{{\left(\rho c_{p}\right)}_{1s}}{{\left(\rho c_{p}\right)}_{f}}\right)+\phi_{2s}\left(\frac{{\left(\rho c_{p}\right)}_{2s}}{{\left(\rho c_{p}\right)}_{f}}\right). \end{array}$$

where the dimensional parameters contained in the equations above are the following: $S=\frac{b{H_{1}}^{2}}{2\nu_{f}},$ $M^{2}=\frac{\sigma_{f}B_{0}^{2}{H_{1}}^{2}}{\rho_{f}\nu_{f}},$ $\lambda =\frac{2{H_{1}}^{2}{\left(1-bt\right)}^{2}g\beta_{f}\left(T_{w}-T_{h_{1}}\right)}{br_{1}\nu_{f}},$ $R_{d}=\frac{4\sigma^{*}T_{h_{1}}^{3}}{k^{*}k_{f}},$ $\theta_{h_{1}}=\frac{T_{w}}{T_{h_{1}}}$, $A=\frac{w_{0}}{bH_{1}}$, and $\mathrm{Pr}=\frac{\nu_{f}}{\alpha_{f}}$ while these parameters are namely called the squeeze number, Hartmann number or magnetic, mixed convective parameter, radiation parameter, temperature ratio parameter, the blowing/suction parameter, and the Prandtl number, respectively.

## Engineering Quantities of Interest

The skin friction factor and local Nusselt number are signified as:

(14) $$\begin{array}{l} C_{Fr_{1}}=\frac{{\tau_{r_{1}z_{1}}|}_{z_{1}=h_{1}\left(t\right)}}{\rho_{f}{\left(\frac{-bH_{1}}{2\sqrt{1-bt}}\right)}^{2}},Nu_{r_{1}}=\frac{H_{1}q_{w}}{k_{f}\left(T_{w}-T_{h_{1}}\right)}, \end{array}$$

where

(15) $$\begin{array}{l} \tau_{r_{1}z_{1}}=\mu_{hnf}{\left(\frac{\partial u_{1}}{\partial z_{1}}+\frac{\partial w_{1}}{\partial r_{1}}\right)|}_{z_{1}=h_{1}\left(t\right)},\\ \;q_{w}={-k_{hnf}\left(\frac{\partial T_{1}}{\partial z_{1}}\right)+{\left(q_{r_{1}}\right)}_{w}|}_{z_{1}=h_{1}\left(t\right)}. \end{array}$$

In terms of dimensional form, the local skin friction and Nusselt number are

(16) H12r12Rer1CFr1=μhnfμfF″(1),1-btNur1=-(khnfkf+43Rd(1+(θh1-1)θ(1))3)θ′(1),

where Rer1=r1bH11-bt2νf is the local Reynolds number.

## Numerical Procedure

The higher-order Equations (11) and (12) along with the boundary restriction (13) are a highly non-linear dimensional form of ODEs in nature. The solution of these equations is very difficult to obtain analytically. Therefore, we utilized the numerical approach of bvp4c, which is based on the three-stage Lobatto 3A method. This process is fruitful to handle the one-point problem in the first order. In broad mode, Lobatto 3A techniques have been exercised for the two-point boundary value problem due to their very exceptional properties of stability and the fact that they contain accuracy of fourth order over the entire interval. To execute this approach, the equations in the dimensional form are exercised owing to new variables and converted into the first-order system of equations. To do the technique as outlined, let

(17) $$\begin{array}{l} F=B_{1}^{*},F^{\prime }=B_{2}^{*},F^{\prime \prime }=B_{3}^{*},F^{\prime \prime \prime }=B_{4}^{*},\theta =B_{5}^{*},\theta^{\prime }=B_{6}^{*}, \end{array}$$

Plugging Equation (17) in the dimensional form of ODEs to get the first-order set of ODEs as follows:

(18) $$\begin{array}{l} \left(\begin{array}{c} B_{1}^{*\prime }\\ B_{2}^{*\prime }\\ B_{3}^{*\prime }\\ B_{4}^{*\prime }\\ B_{5}^{*\prime }\\ B_{6}^{*\prime } \end{array}\right)\\ =\left(\begin{array}{c} B_{2}^{*}\\ B_{3}^{*}\\ B_{4}^{*}\\ \frac{SA_{1}}{A_{2}}\left(3B_{3}^{*}+\eta B_{4}^{*}+2B_{1}^{*}B_{4}^{*}\right)+\frac{A_{3}M^{2}}{A_{2}}B_{3}^{*}-\frac{\lambda A_{4}}{A_{2}}B_{6}^{*}\\ B_{6}^{*}\\ \frac{\left\{-4R_{d}{\left(1+\left(\theta_{h_{1}}-1\right)B_{5}^{*}\right)}^{2}\left(\theta_{h_{1}}-1\right){\left(B_{6}^{*}\right)}^{2}+\mathrm{Pr}SA_{6}\left(\eta B_{6}^{*}-2B_{1}^{*}B_{6}^{*}\right)\right\}}{\left(A_{5}+\frac{4}{3}R_{d}{\left(1+\left(\theta_{h_{1}}-1\right)B_{5}^{*}\right)}^{3}\right)} \end{array}\right). \end{array}$$

Subject to corresponding initial conditions

(19) $$\begin{array}{l} \left(\begin{array}{c} B_{1}^{*}\left(0\right)\\ B_{1}^{*}\left(1\right)\\ B_{2}^{*}\left(0\right)\\ B_{2}^{*}\left(1\right)\\ B_{5}^{*}\left(0\right)\\ B_{5}^{*}\left(1\right) \end{array}\right)=\left(\begin{array}{c} A\\ 0.5\\ 0\\ 0\\ 1\\ 0 \end{array}\right). \end{array}$$

To carry out this procedure, we have to choose the finite value of the boundary η → ∞, say η _∞_ and the computated value of this constraint depends on the other governing constraints involved in the system of the first-order ODEs. The aforementioned Equation (19) contains one unknown *A*, which is constant, and the value depends on the other constraints that influenced the system. In the current computation process, the value of the unknown constant *A* is fixed to be 0.2, while the other involved sundry constraints whose numerical fixed values are mentioned in the portion of Results and Discussion. Thus, to run the process of bvp4c, it required some initial early guesses that satisfied Equation (19). Moreover, the problem is bounded between 0 and 1. So, in the computation, the value of the step size is reserved as Δη = 0.01. The iterative process is repeated, waiting for the required tests to be obtained in compliance with the convergence criterion up to the accuracy point 10^−10^.

## Results and Discussion

The physical trend of the engaged parameters on the temperature distribution and nanofluid velocity was investigated. During the computation, it allocated that the pertaining parameters be fixed as ϕ = 0.03, *M* = 0.3, *R*_*d*_ = 0.5, θ_*h*_1__ = 0.5, *S* = 0.5, *A* = 0.2, and λ = 0.5, except for the values depicted in the tables and graphs. Table 2 illustrates the validation of the current outcomes of H12r12Rer1CFr1 with Hayat et al.'s (2012a) existing results. It confirmed the outstanding harmony with available results. Tables 3, 4 are set to scrutinize the behavior of the physical parameters in the form of the values of H12r12Rer1CFr1 and $\sqrt{1-bt}Nu_{r_{1}}$ in the opposing and assisting flows. The friction factor and heat transport rate decline owing to *M* in the assisting flow and augment in the opposing flow. Whereas, the ferroparticle volume fraction tremendously augments the heat transport rate and friction factor in the assisting flow, a divergent performance is seen in the opposing flow. Physically, the greater amount of ϕ creates a larger energy conveyed via the fluid flow connected with the unbalanced progress of the ultrafine materials and thus generates a significant improvement in the friction factor and the heat transport rate. The squeezed parameter augments the friction factor and the heat transport rate in both flows.

**Table 2:** Justification of the outcomes for H12r12Rer1CFr1 when *R*_*d*_ = θ_*h*_1__ = λ = ϕ = 0.

***A***	***S***	***M***	**Hayat et al. (2012a)**	**Present**
−0.1	0.1	0.1	−3.62306	−3.61948
0.0			−3.01553	−3.01611
0.1			−2.40948	−2.41001
0.3			−1.20180	−1.20180
0.4			−0.60016	−0.60011

**Table 3:** The numerical values of H12r12Rer1CFr1 and $\sqrt{1-bt}Nu_{r_{1}}$ for the assisting flow λ = 0.5 when *R*_*d*_ = θ_*h*_1__ = 1, *A* = 0.2.

***M***	**ϕ_1_, ϕ_2_**	***S***	**$\frac{{H_{1}}^{2}}{{r_{1}}^{2}}{\text{Re}}_{r_{1}}C_{Fr_{1}}$**	**$\sqrt{1-bt}Nu_{r_{1}}$**
0.5	0.03	0.5	4.1776246	5.8056969
1.0			4.1691178	5.794217
1.5			4.1611946	5.7831014
2.0			4.1538254	5.7723334
0.5	0.03	0.5	4.1776246	5.8056969
	0.07		7.0756704	7.3191953
	0.1		9.0887832	8.5698581
	0.15		11.949806	10.806558
0.5	0.03	0.5	4.1776246	5.8056969
		01	4.2184234	10.162559
		1.5	4.1255412	15.045831
		02	4.0009439	20.243336

**Table 4:** The numerical values of H12r12Rer1CFr1 and $\sqrt{1-bt}Nu_{r_{1}}$ for the opposing flow λ = −0.5 when *R*_*d*_ = θ_*h*_1__ = 1, *A* = 0.2.

***M***	**ϕ_1_, ϕ_2_**	***S***	**$\frac{{H_{1}}^{2}}{{r_{1}}^{2}}{\text{Re}}_{r_{1}}C_{Fr_{1}}$**	**$\sqrt{1-bt}Nu_{r_{1}}$**
0.5	0.03	0.5	−0.64438756	4.0589601
1.0			−0.6057098	4.0731655
1.5			−0.56780399	4.0869506
2.0			−0.53063898	4.1003344
0.5	0.03	0.5	−0.64438756	4.0589601
	0.07		−3.0922418	3.0372273
	0.1		−4.188812	2.3968991
	0.15		−4.8559347	1.7439673
0.5	0.03	0.5	−0.64438756	4.0589601
		01	−0.55996244	6.5819513
		1.5	−0.42770728	10.49348
		02	−0.20791833	15.892787

**Figure 2:**
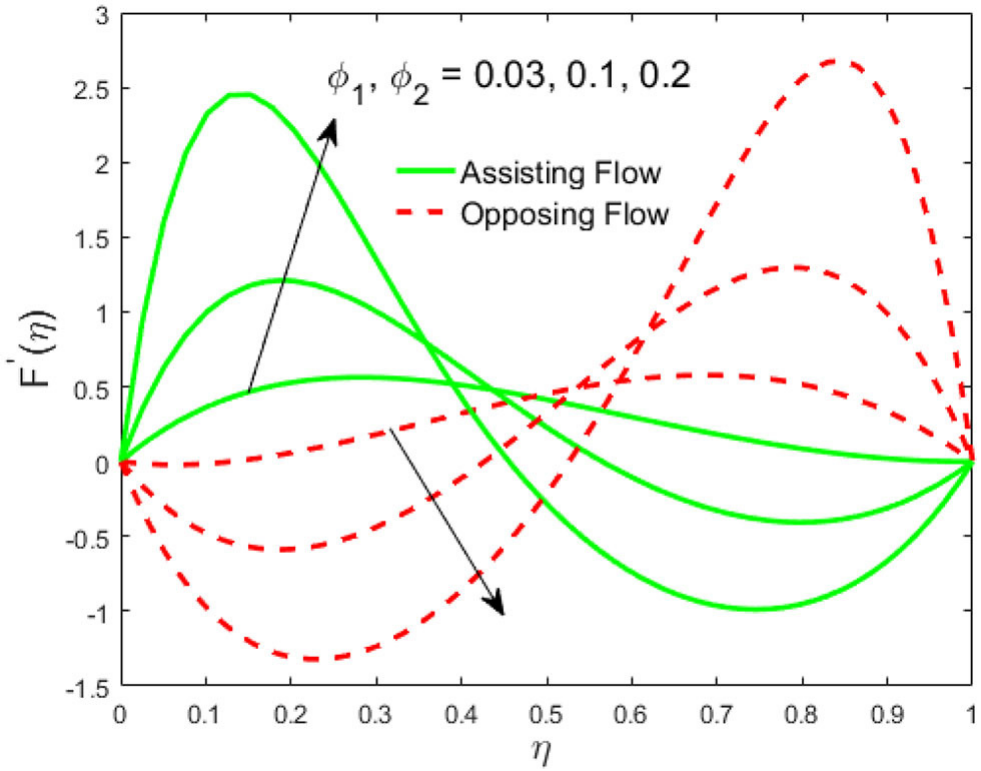
Impact of ϕ_1_, ϕ_2_ on *F*′(η).

**Figure 3:**
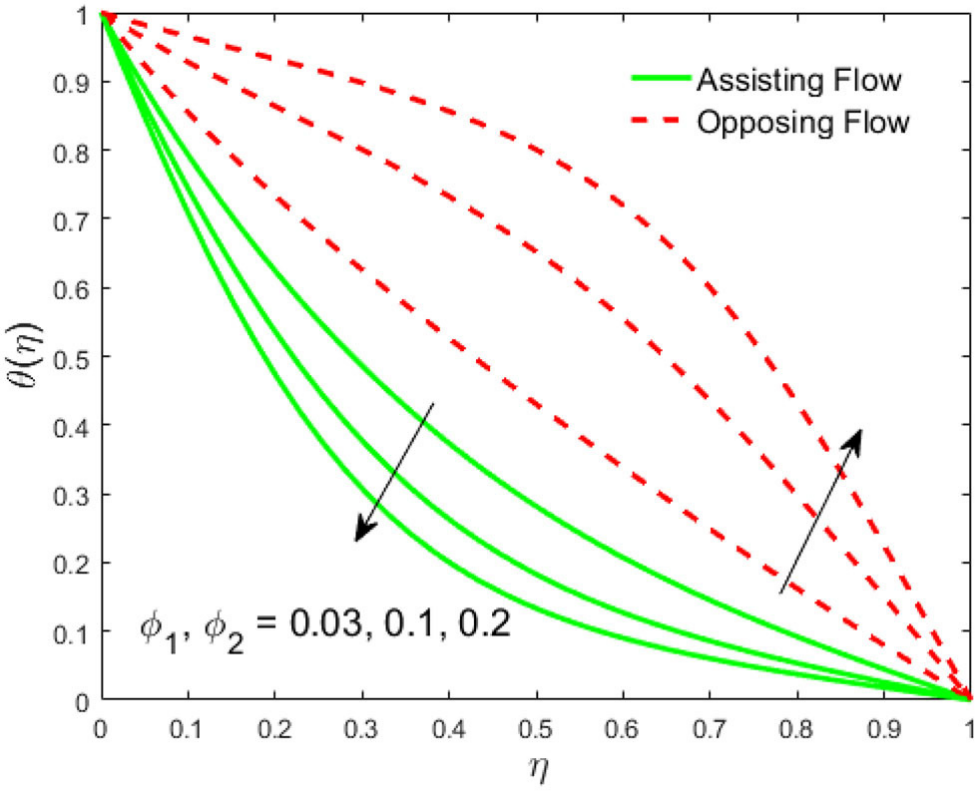
Impact of ϕ_1_, ϕ_2_ on θ(η).

**Figure 4:**
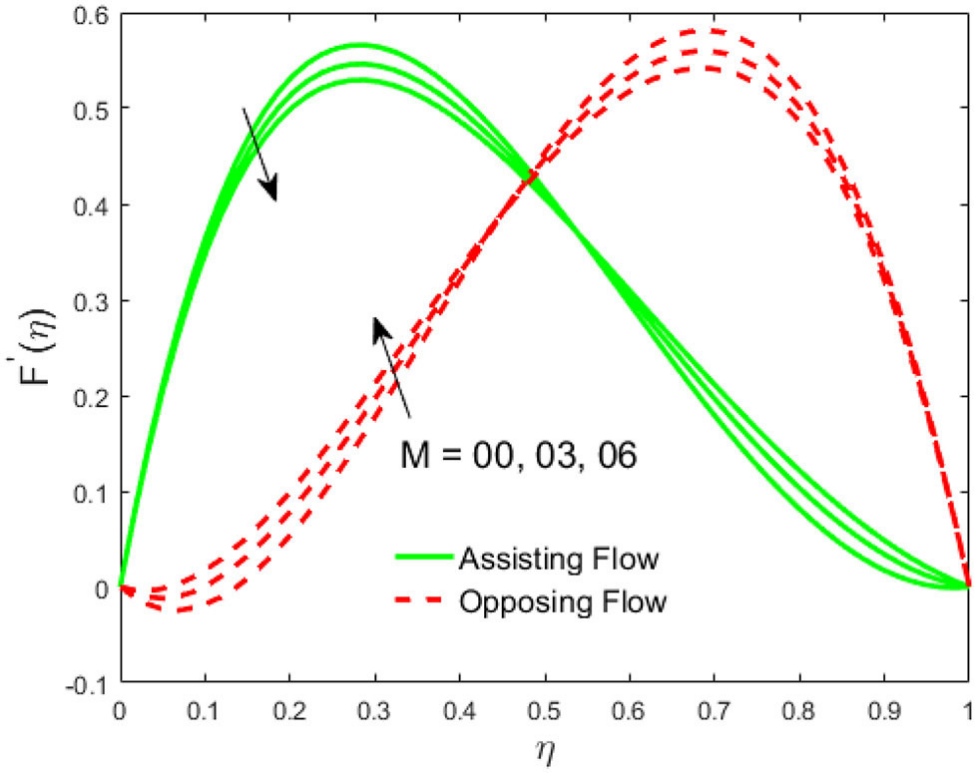
Impact of *M* on *F*′(η).

**Figure 5:**
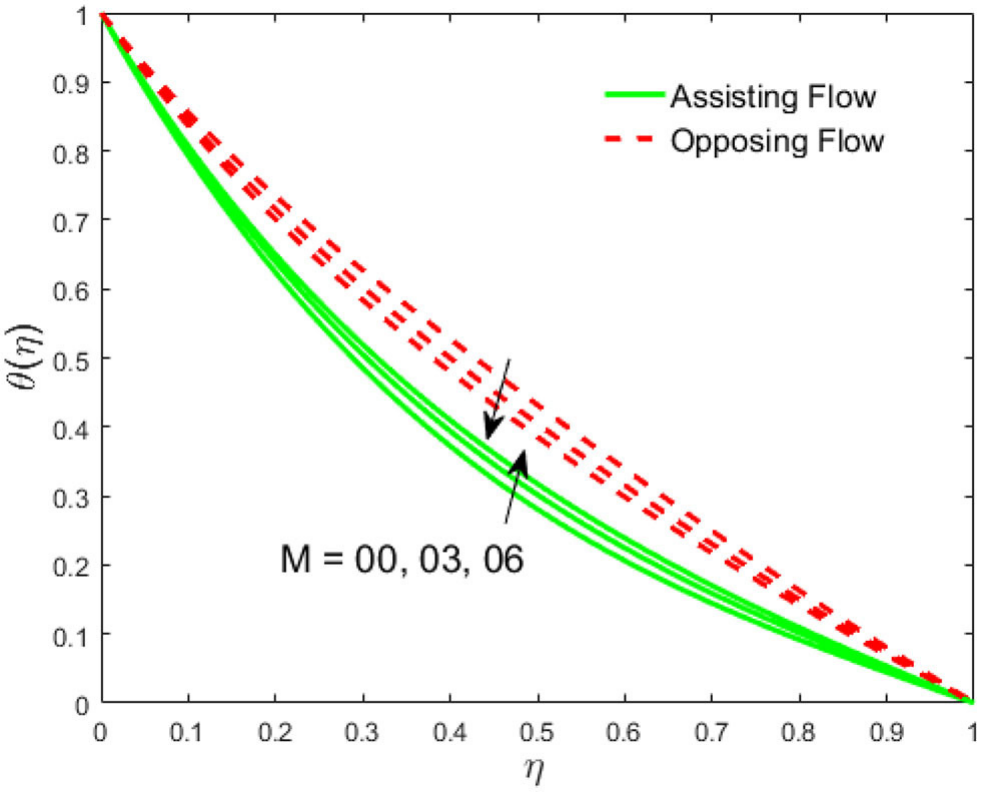
Impact of *M* on θ(η).

**Figure 6:**
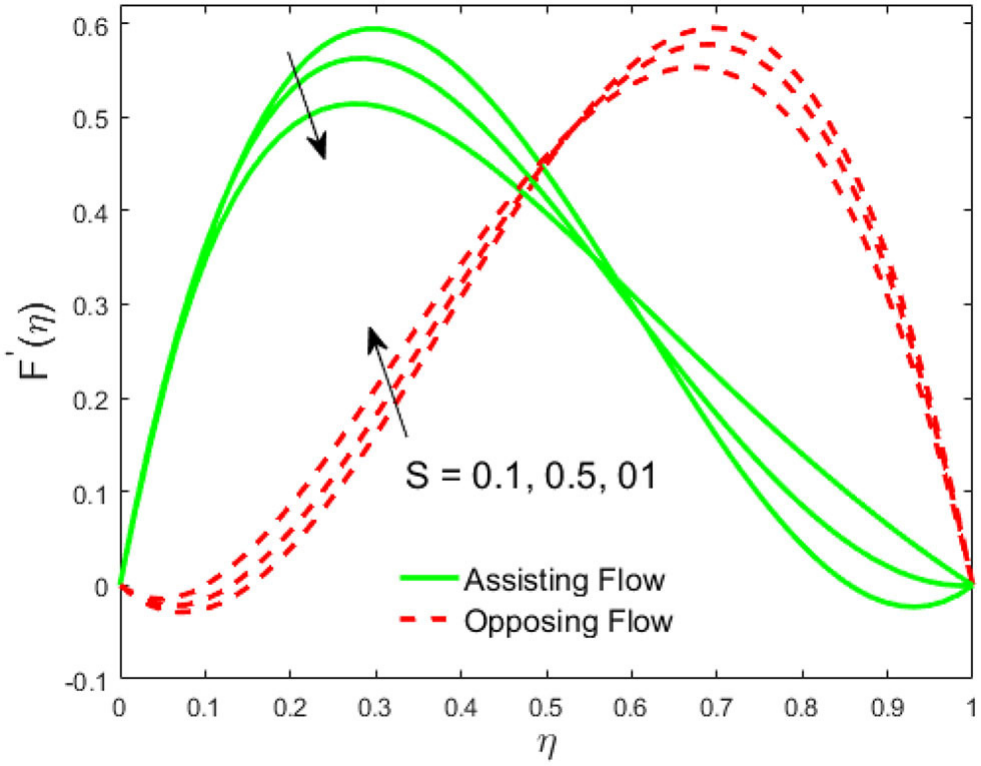
Impact of *S* on *F*′(η).

**Figure 7:**
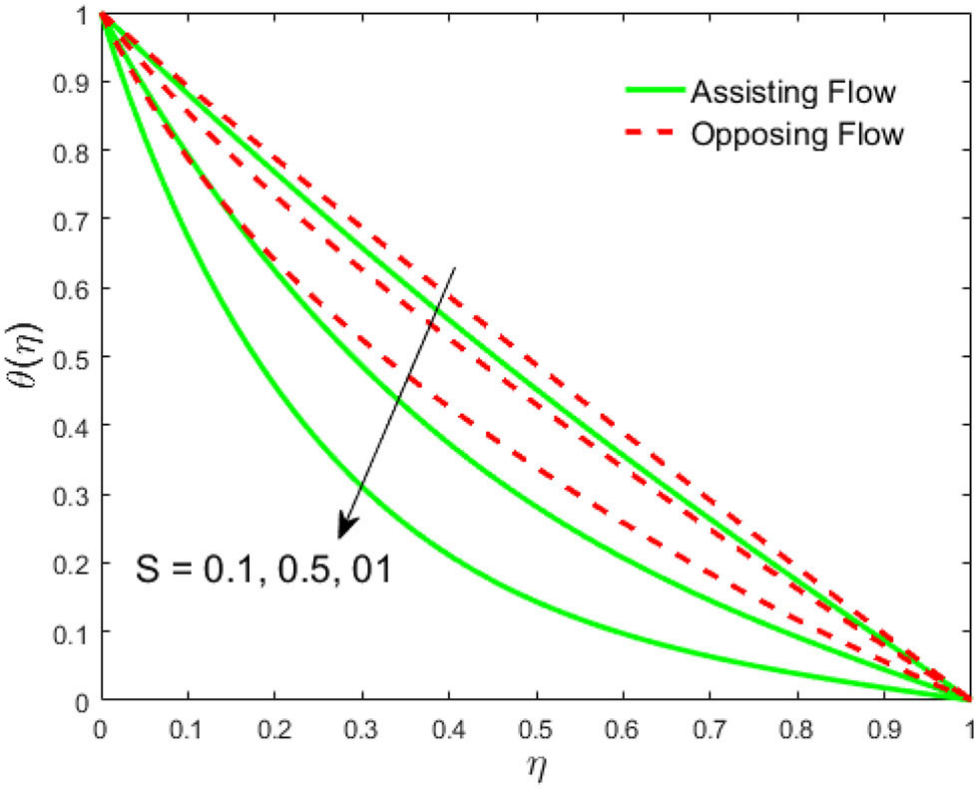
Impact of *S* on θ(η).

**Figure 8:**
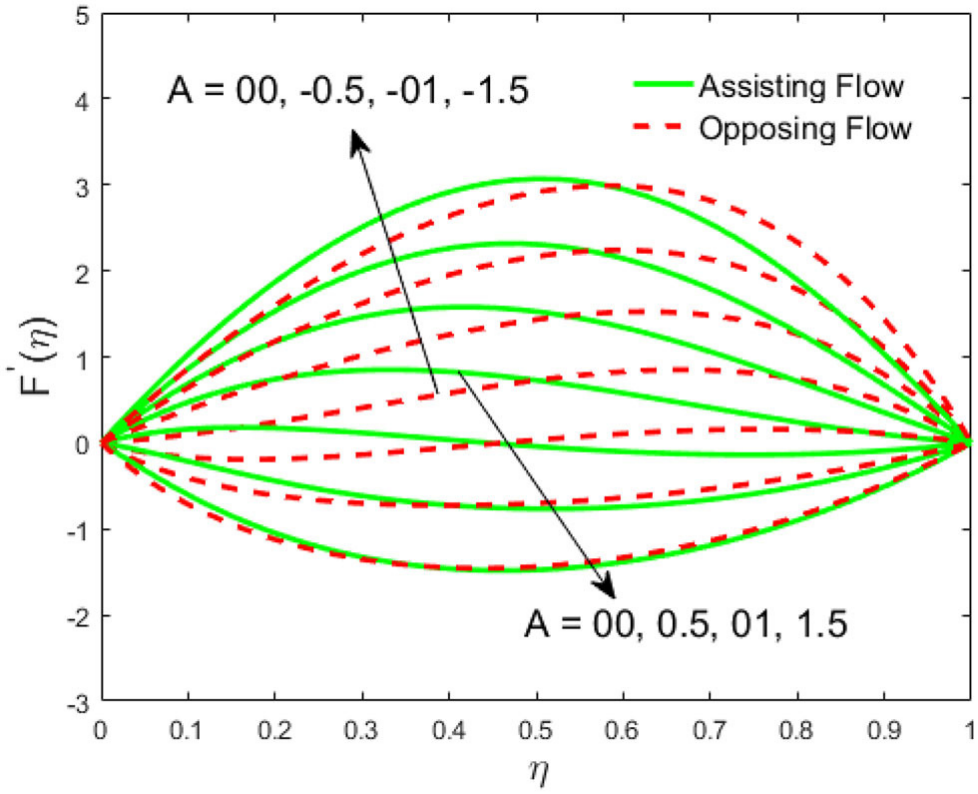
Impact of *A* on *F*′(η).

**Figure 9:**
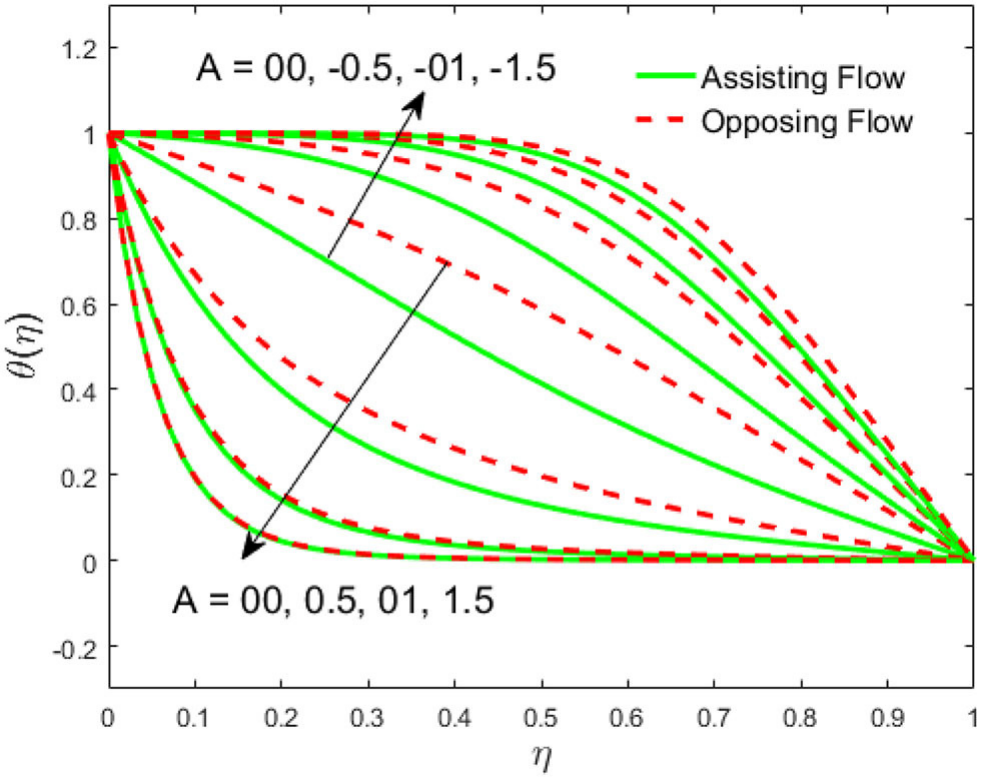
Impact of A on θ(η).

**Figure 10:**
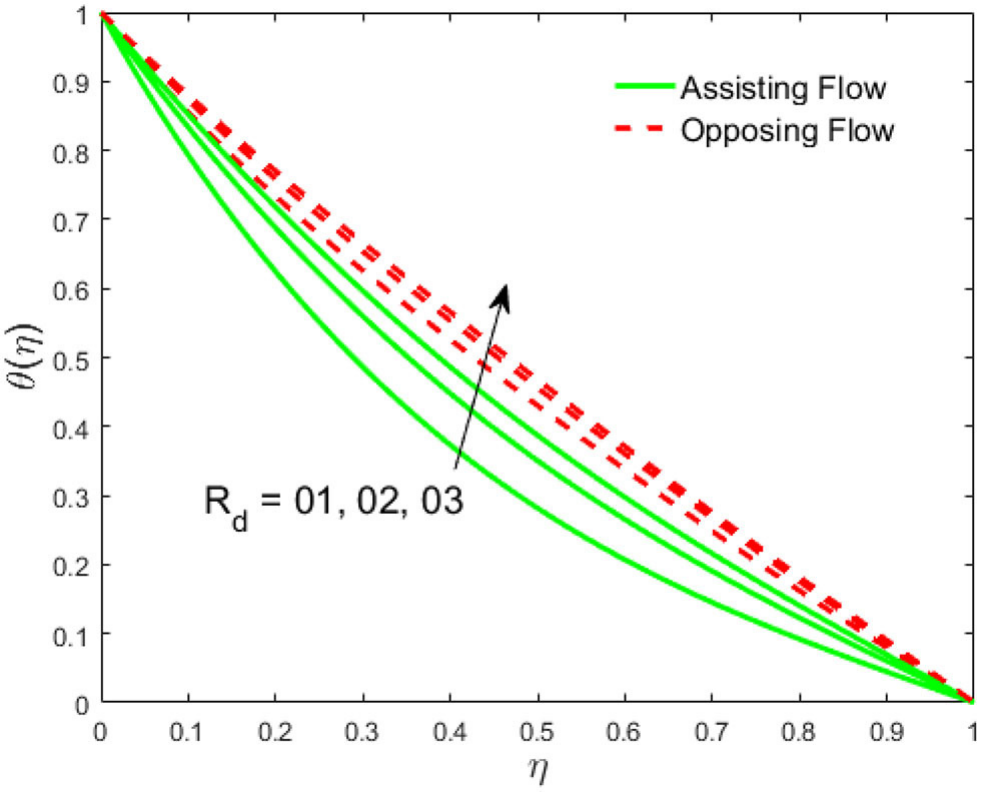
Impact of R_d_ on θ(η).

**Figure 11:**
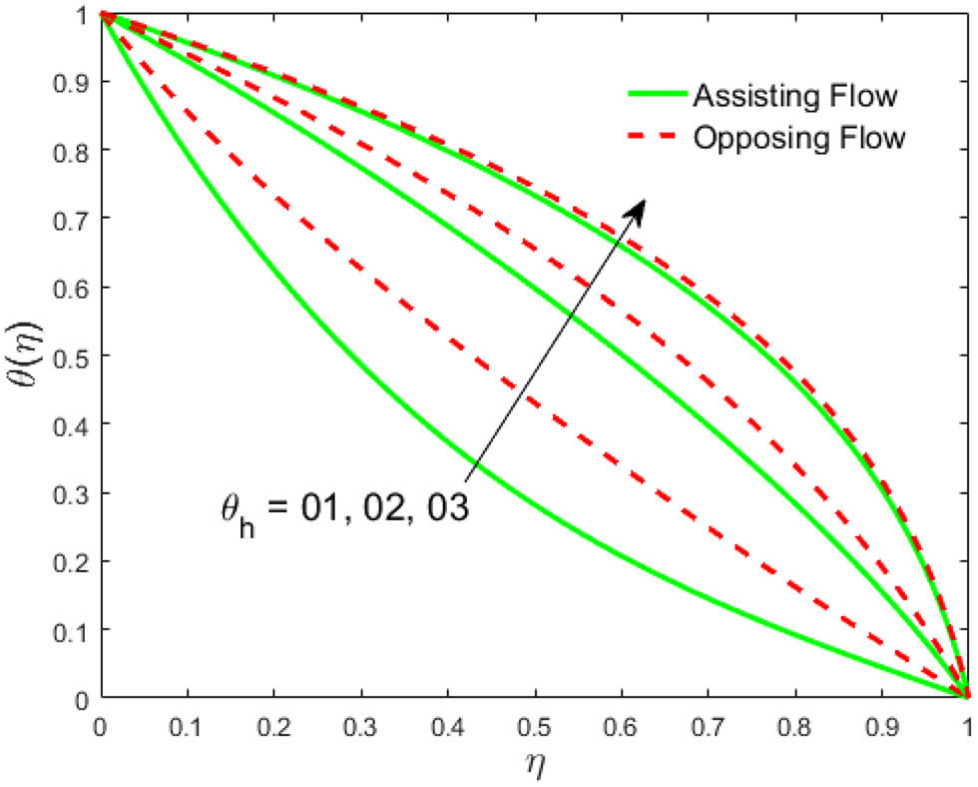
Impact of *R*_*d*_ on θ(η).

**Figure 12:**
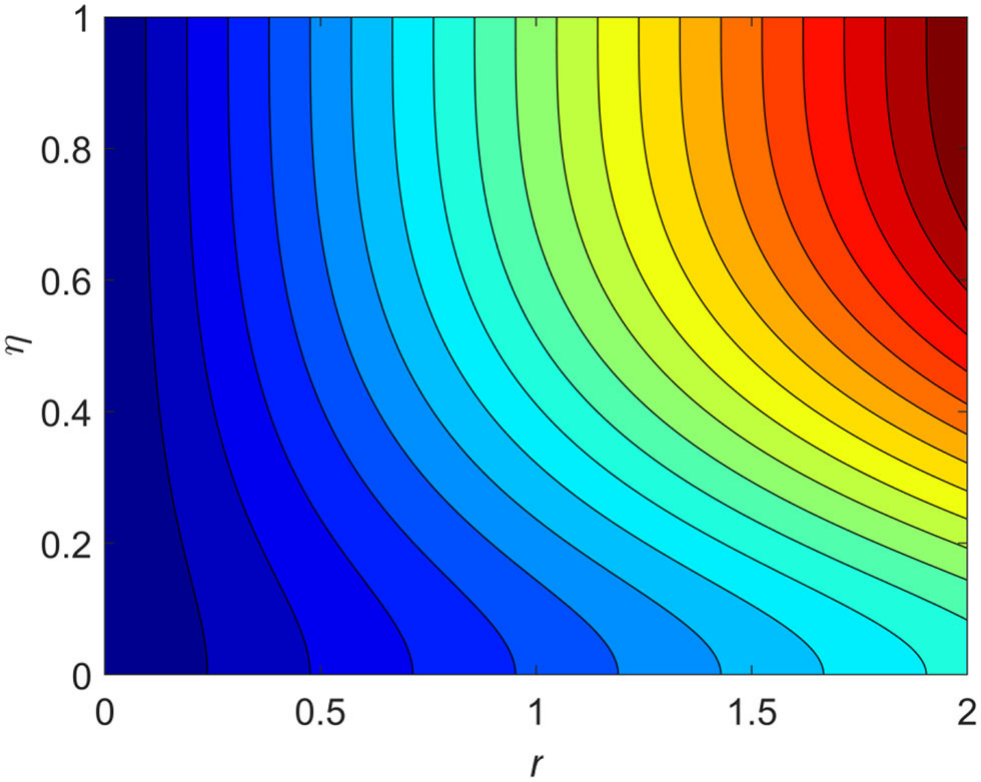
Streamline patterns for suction *A* = 0.2 > 0.

**Figure 13:**
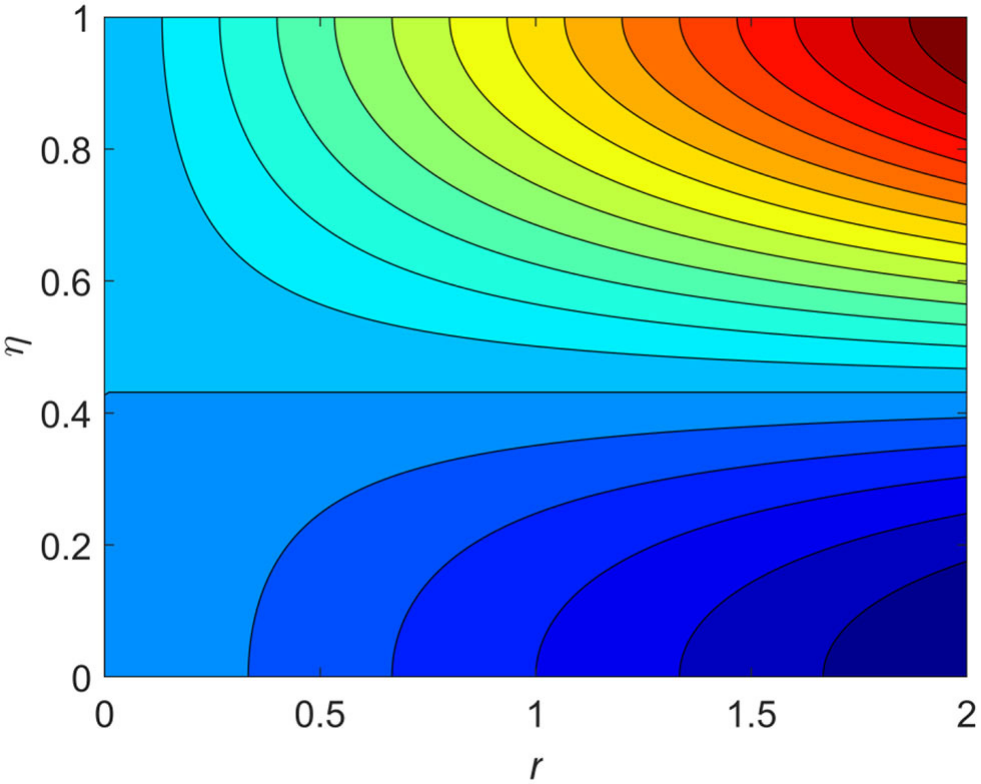
Streamline patterns for blowing *A* = −0.2 < 0.

The influence of the volume fraction of hybrid ferrofluid (ϕ) on the fluid velocity (*F*′(η)) and temperature (θ(η)) for the assisting flow (ASF) and the opposing flow (OPF) is revealed in Figures 2, 3, respectively. Figure 2 portrays that the fluid velocity increases for the ASF and decreases for the OPF as we augmented the values of (ϕ) simultaneously up to the approximate range of η_max_ = 0.3 and η_max_ = 0.58, respectively, while the reverse behavior of the velocity is seen in both the ASF and the OPF when the similarity variable is greater than the aforementioned ranges. The solution in the opposing flow is more dominant as compared to the assisting flow in the velocity profile. This is apparent from reality that a greater amount of (ϕ) communicates to increase the ferrofluid thermal conductivity, which inspires the diffusion of heat in order for the heat to impulsively disperse near the surface of the disks. Figure 3 demonstrates the temperature distribution for the ASF as well as the OPF against the similarity variable for the hybrid ferrofluid (ϕ). The temperature distribution diminishes in the ASF and upsurges in the OPF as we boost up the value of the hybrid ferrofluid (ϕ). The large amount of (ϕ) generates a great energy conveyed during the flow connected with the uneven progress of the ultrafine materials and hence produces a substantial improvement in the rate of heat transport that consequently augments the temperature. It is fascinating to see in the figure that the thermal boundary layer in the ASF is less extensive than the OPF. Figures 4, 5 show the Hartmann impact *M* on (*F*′(η)) and (θ(η)) against the similarity variable η for the phenomenon of both the ASF and OPF, respectively. In case of assisting flow, the fluid velocity reduces as we move along the horizontal axis within the range of η_max_ = 0.51, and in the same spotted range, the velocity is enhanced in the opposing flow for the continuous positive change in the values of *M*, while for the simultaneous cases, the opposite behavior is, respectively, shown as we move out of the aforementioned range. Further, it is scrutinized that the liquid velocity is maximum attained for the ASF than that of OPF. Figure 5 exhibits that the temperature distribution grows higher in the ASF and decreases in the OPF for the regular augmentation in the parameter *M*. More generally, the temperature behavior of the fluid is enhanced in the ASF due to the higher values of *M*. The constant augmentations in *M* will reduce the thickness of the thermal boundary layer of the fluid regularly. Physically, the escorting hybrid ferrofluid produces a sort of resistive force, that is to say, the Lorentz force. Together with this, the flow generates a certain amount of heat energy. It is worth mentioning that the hybrid ferroliquid is extremely prejudiced through the drag force in the ASF compared with OPF. The impact of the squeeze number *S* for both the cases of heating and cooled surfaces on the velocity and the distribution of temperature is plotted in Figures 6, 7, respectively. Here, we have taken the values of squeeze number to be greater than zero, which physically shows that the upper disk is moving away from the lower stationary disk, while in the case of opposite inequality of the squeeze number, corresponding to the lower disk moving away from the upper disk, this was not discussed graphically. More precisely, Figure 6 shows that the velocity is declining against the dimensionless variable η in the domain from 0 ≤ η ≤ 0.6 for the ASF when we improve the value of *S*, while the flow velocity is jumping up in the same domain for the OPF. When we altered the domain from 0.6 ≤ η ≤ 01, then the opposite behavior (like 0 ≤ η ≤ 0.6) for the flow velocity is accomplished. Despite this, the momentum boundary layer is lower in the OPF when we compared with the ASF. The variation in the temperature distribution is shown in Figure 7, where the distribution in the temperature of the fluid flow is consistently falling as we augment the value of *S* for both the ASF and OPF. It is interestingly seen in the pictures that both the thermal boundary layer and the distribution in the temperature are declining. The last profile of velocity is behaviorally exposed in Figure 8 for suction *A* > 0 and blowing *A* < 0. The value of the velocity decreases in both the ASF and OPF for the four selected values of (*A* = 0, 1, 1.5, 2) while the opposite trend of the velocity is achieved in the case of injection (*A* = 0, −1, −1.5, −2). In contrast to suction, the momentum boundary layer is more dominant in the injection case. The impact of the blowing/suction parameter *A* in the temperature profile against the new variable η for both the positive and negative values of λ is depicted in Figure 9. A very similar behavior is observed (such as in Figure 8), but the temperature and thermal boundary layer continue to fall due to the parameter value of (*A* = 0, 1, 1.5, 2) as compared to the parameter in the case of injection (*A* = 0, −1, −1.5, −2). Figures 10, 11 display the stimulus of the parameters *R*_*d*_ and θ_*h*_1__ on (θ(η)) for the assisting and opposing flows, respectively. Figure 10 shows the increasing behavior of temperature in the ASF and in the OPF for the greater value of *R*_*d*_. However, it is widely known that its radiation process is just the heat transfer phenomenon that emits the energy through fluid particles so that an extra heat is generated in the flow. Thus, it is identified as a development in the thermal boundary layer for greater *R*_*d*_. Behavior-wise, one can get a similar temperature distribution (like in Figure 10), which is highlighted in Figure 11. We noticed very motivating results in that the heat curves θ(η) are further exaggerated in the impact of radiation parameter when we compared with the temperature ratio parameter. Finally, Figures 12, 13 are sketched to observe the behavior of streamlines for suction and blowing. These patterns suggest that streamlines' behavior in case of suction is smooth, while in the case of blowing, the streamlines are divided into two regions.

## Conclusion

This research succeeded in conferring the exploration of the mixed convective squeezed flow of hybrid ferroparticles within the two discs under the impacts of MHD and radiation. Water + ethylene 50%:50% is taken as a base liquid of the channel. The leading equations are resolved through the Lobatto 3A formula. To ensure the correctness of the outcomes, an assessment of existing results is made. The influences of numerous significant constraints on the fluid flow are consummated via graphs. The following significant outcomes are summarized:

- The velocity of hybrid nanofluid upsurges due to ϕ in ASF and decelerates in OPF, while the temperature declines in ASF and augments in OPF.
- The Hartmann number upsurges and shrinks the temperature, respectively, in ASF and OPF, while the opposing trend is seen in the velocity.
- The squeezed number declines the temperature in both flows and augments the velocity in the OPF and shrinks that in the ASF.
- The suction parameter augments the velocity and temperature, while the injection shrinks the velocity and temperature in both flows.

- The radiation and temperature ratio parameters upsurge the temperature in both flows.
- The friction factor and heat transport rate decline due to *M* in the ASF and augment in the OPF.
- The ferroparticle volume fraction tremendously augments the friction factor and the heat transport rate in the ASF, while the opposing behavior is seen in the OPF.

In the end, this work can be further considered by taking hybrid nanoparticles with different shape factors along with mass transfer. Also, this problem may be extended into the two-phase model by taking into account the Buongiorno model.

## Data Availability Statement

All datasets generated for this study are included in the article/supplementary material.

## Author Contributions

UK did formulation and transformed the problem. AZ computed results. IK, KN, and DB wrote the manuscript. DB, IK, and KN revised the manuscript. All authors contributed to the article and approved the submitted version.

## Conflict of Interest

The authors declare that the research was conducted in the absence of any commercial or financial relationships that could be construed as a potential conflict of interest.

## Nomenclature

**Table:**

*A*	Suction/blowing parameter
*b*	Squeezing rate
*B*_0_	Magnetic field strength
*C*_*F*_*r*__1__	Skin friction coefficient
*c*_*p*_	Specific heat (J/kg K)
*H*_1_	Initial point of the disk
*F*	Dimensionless velocity
*g*	Gravity acceleration (m^2^/s)
*p*	Pressure
*k*	Thermal conductivity (W/mK)
*M*	Hartmann number
*R*_*d*_	Radiation parameter
*Nu*_*r*_1__	Nusselt number
Pr	Prandtl number
Re*r*_1_	Local Reynolds number
*S*	Squeeze number
*T*_1_	Temperature (K)
*T*_*w*_	Constant temperature of the lower disk (K)
*T*_*h*_1__	Constant temperature of the upper disk (K)
θ	Dimensionless temperature
θ_*h*_1__	Temperature ratio parameter
(*u*_1_, *w*_1_)	Velocity components (m/s)
*w*_*w*_	Mass flux velocity
*w*_0_	Constant
(*r*_1_, θ_1_, *z*_1_)	Cylindrical coordinates
*t*	Time
Greek Symbols	
α	thermal diffusivity (m^2^/s)
β	Thermal expansion (K^–1^)
λ	Mixed convection parameter
μ	dynamic viscosity (kg/ms)
ϕ_1_, ϕ_2_	The volume fraction of the individual ferroparticles
*v*_*f*_	Kinematic viscosity of the base fluid (m^2^/s)
ρ	Density (kg/m^3^)
σ	The electrical conductivity (S/m)
σ*	Stefan Boltzmann constant (kg/s^3^ K^4^)
*k**	Absorption coefficient
τ	Dimensionless variable
η	Similarity variable
Subscripts	
*f*	Base fluid
*s_1_,s_2_*	Solid particles
*hnf*	Hybrid nanofluid
*w*	Wall boundary condition
*h*_1_	Height between the two disk
Superscripts	
‘	Derivative w.r.t. η
